# A Complexity Lens on the COVID-19 Pandemic

**DOI:** 10.34172/ijhpm.2021.55

**Published:** 2021-05-26

**Authors:** Didier Wernli, Fabrizio Tediosi, Karl Blanchet, Kelley Lee, Chantal Morel, Didier Pittet, Nicolas Levrat, Oran Young

**Affiliations:** ^1^Geneva Transformative Governance Lab, Global Studies Institute, University of Geneva, Geneva, Switzerland.; ^2^School of Public Health, Li Ka Shing Faculty of Medicine, University of Hong Kong, Hong Kong, China.; ^3^Swiss Tropical and Public Health Institute, University of Basel, Basel, Switzerland.; ^4^Geneva Centre of Humanitarian Studies, Faculty of Medicine, University of Geneva and Graduate Institute of International and Development Studies, Geneva, Switzerland.; ^5^Faculty of Health Sciences, Simon Fraser University, Burnaby, BC, Canada.; ^6^Infection Control Programme, University of Geneva Hospitals and Faculty of Medicine, Geneva, Switzerland.; ^7^Bren School of Environmental Science and Management, University of California at Santa Barbara, Santa Barbara, CA, USA.

## Introduction

The most striking feature of the coronavirus disease 2019 (COVID-19) pandemic and associated responses is its social and ecological complexity. Applying a complexity lens can improve our understanding of the current COVID-19 pandemic but how can this best be done? Complexity science is not a unified theory but rather a collection of concepts, theories, and methods that are increasingly influencing a range of scholarly disciplines. Complex systems can be simply defined as “co-evolving multilayer networks.”^1^ This definition stresses the dynamic nature of causality as well as the emergent and difficult to predict behaviour in networks that can adapt to a changing environment. Based on this definition, we describe key features of the COVID-19 pandemic, draw insights from complexity science about the nature of these features, and understand the implications for effective response and governance. This framework offers a relevant approach for shaping future research on the social ecological impact of the pandemic including comparative measures of resilience of different health systems to future events.

## Complex Causality: Understanding the Drivers of and Vulnerabilities to the Crisis

At the core of complexity theory is the idea that elements interacting in a system result in a behaviour or an outcome that is more than the sum of the parts. The emergence and global spread of severe acute respiratory syndrome coronavirus 2 (SARS-CoV-2) resulted from multilevel and multiscale interactions between host, pathogen, and other factors at the human, animal, and environment interface. Beyond the study of infectious diseases spillover, complex causality draws our attention to the broader vulnerabilities that relate to virus transmission and effects of the pandemic. Global travel obviously resulted in the rapid transmission of SARS-CoV-2 across the world, but factors as diverse as population health status, quality of institutions, and political leadership and philosophy have affected the ability of countries to mount effective responses. For example, the austerity policies adopted in the wake of the 2008 financial crisis led many high-income countries to under-invest in health systems, making them, in turn, more vulnerable during the COVID-19 pandemic.^2^ Socioeconomic inequities within and between populations have led to differences in how the virus has played out, drawing attention to the neglect of political and social determinants of health. A better understanding of how diverse and interacting contextual factors result in vulnerabilities and capabilities is an essential starting point for future preparedness.^3^

## Nonlinear Propagation: How Things Can Get Out of Control

COVID-19 exhibits several processes of contagion in complex networks. It has been quickly revealed that the transmission of SARS-CoV-2 follows a power law distribution whereby some individuals cause most subsequent cases.^4^ This understanding led some countries such as Japan to rapidly prioritize strategies that target close contact in crowded and enclosed settings. Yet the phenomenon of contagion has not been limited to the virus. Network theory applied to human behaviour tells us that complex contagion requires contact with multiple sources of reinforcement to be transmitted.^5^ This is typical of the diffusion of social norms, for instance, which has led to the rapid adoption of new behaviours to control COVID-19. Conversely, widespread disinformation can amplify the spread of the virus, for example by encouraging inappropriate behaviours.^6^ Understanding contagion through a complexity lens may thus help design strategies to counter or mitigate disinformation, and support continued efforts by public health authorities to promote scientific evidence.^7^ Overall, a fuller understanding of the dynamics of contagion demonstrates the need for rapid action when a new pathogen emerges. Such action, in turn, requires a governance model that can adapt quickly. Several countries in East Asia as well as Australia and New Zealand endorsed a go-fast and go-early strategy that allowed them to control community transmission based on an understanding of social connectivity. The success of these ‘zero COVID-19’ strategies have also been the basis for proposed strategies for safe reopening in Europe and elsewhere.^8^

## Systemic Effects Beyond Contagion: Balancing the Impact of Action and Inaction

COVID-19 is proving to have system-wide effects far beyond health systems. These effects, which often result from reinforcing feedback processes, have been particularly manifest in countries that have been less effective at controlling the pandemic. System-wide effects are likely when insufficient action to control the virus results in increased fear and public distrust. In Brazil, for example, COVID-19 is severely disrupting the health system but is also undermining economic, social and political systems.^9^ However, policies to tackle COVID-19 such as lockdowns adopted in many countries, particularly when they are long lasting and repetitive, can also have unintended consequences. For example, when measures prevent people from earning a living, which leads to major hardship in countries lacking sufficient social safety nets,^10^ can also erode public confidence. Policy failures may remain isolated from each other but, when a public health emergency impacts multiple systems, a particularly dangerous process is cascading failure ie, when the failure of one component leads to failures of other interconnected components. The resulting amplification leads to outcomes that are difficult to predict and control.^11^ To prevent cascading economic failure, countries have been relatively quick to implement countermeasures. For example, the United States has adopted stimulus packages amounting to several trillion US dollars. Overall, systemic effects call for governance processes that build upon different perspectives and manage interplay between different goals and sectors.^12^

## Resilience: The Role of Multiple Agents in the Response

COVID-19 has tested the capacities of health systems and societies to absorb shock. Resilience stresses the capacities of systems to cope, adapt and transform in the face of disturbances.^13^ In healthcare, health facilities adopted new procedures to isolate COVID-19 patients and to deliver routine healthcare. Many countries reorganized their healthcare system to augment the capacity of hospitals to treat the surge of patients requiring treatment for COVID-19.^14^ In public health, a trial-and-error approach has supported more geographically and timely targeted interventions, especially when coupled with extensive testing and tracing as rapidly implemented in many East Asian countries. In the private sector, some pharmaceutical companies redirected their investment towards the development of health technologies such as vaccines and diagnostics. Beyond the different components of the health response, resilience also covers adaptation to subsequent disruptions in different sectors (eg, health, economic, education, trade). The adaptive capacity of organizations became apparent when they continued to perform their different economic functions, as illustrated by the rapid pivot by many public and private institutions to online work. Moreover, the current COVID-19 pandemic has also tested individual resilience to a disruption that dramatically reduces social interactions. Overall, societal resilience to the COVID-19 pandemic depends on participation of multiple agents in different sectors of society. The need forcritical capacities for resilience to pandemics, reemphasizes the importance of Universal Health Coverage and the Sustainable Development Goals.

## System Shift and Bifurcation: Reversibility and Long-term Trajectory

The COVID-19 pandemic also exhibits typical non-proportional and path-dependant behaviour of non-linear systems. A system shift describes a major change in a system’s feedback and behaviour resulting in a ‘new normal.’ A threshold was crossed when the initially localized epidemic of COVID-19 became a pandemic through an exponential surge of cases worldwide. However, a successful ‘zero COVID-19’ strategy allowed several countries (eg, China, Australia, New Zealand) to largely shift back early on to the initial ‘normal’ state. By contrast, a ‘living with COVID-19’ suppression strategy by many countries, or ‘negating COVID-19’ in a few countries, resulted in an altered state characterized by severe social, economic and health disruptions (Figure). This altered state has in turn accelerated pre-existing societal trends such as, remote working and green transportation. It also prompts a shift toward authoritarianism in some states as noted in Eastern Europe.^15^ Even a return to pre-pandemic conditions may not lead the affected systems to come back to their original state as they have already settled in a new dynamic equilibrium. At the global level, the long-term trajectory of the pandemic will depend on co-evolutionary dynamics between human behaviour and the virus.^16^ The use of technologies such as vaccines can steer the system toward elimination of the virus. With a lack of global coordination, SARS-CoV-2 is however more likely to become endemic with possible seasonal patterns.^17^ Overall, the risks of undesirable irreversible system shifts call for a better assessment of and preparation for systemic risks in the Anthropocene.

**Figure:**
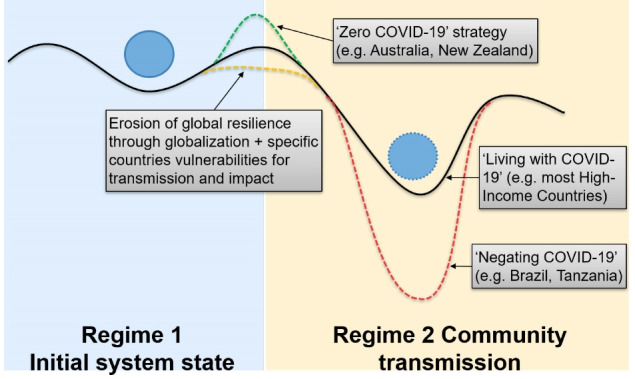
COVID-19 and Systems Shift. Abbreviation: COVID-19, coronavirus disease 2019.

## Conclusion

The COVID-19 pandemic, and its wide-ranging societal impacts, serves as a clarion call for a deeper understanding about how to govern complex social ecological systems.^18^ Events like the COVID-19 crisis cannot be fully understood, much less prevented and dealt with effectively, with our usual arsenal of analytic approaches and tools. An approach emphasizing complex systems not only draws attention to multi-faceted features of the crisis; it also points to critical features of the responses needed to address it.

First, a complexity lens can help policy-makers to grasp the nature of the undergoing systemic crisis and its non-intuitive effects in interlinked systems. Complexity may also strengthen the current narrative at a time of crisis as the basis of effective communication to rally populations around a common cause.^19^

Second, a complexity lens supports collective adaptation and learning in response to the dynamic and constantly evolving crisis. Asserting causality in a complex system remains a major challenge.^20^ To support evidence- and context-based policy, a complexity lens stresses the importance of combining models deriving from evidence from a diverse selection of disciplines.^21^

Third, given the occurrence of surprises in complex systems, a complexity lens shifts strategies from the predictable to the uncertain and unknown.^13^ The challenge is then to adopt policies/governance systems that can reduce the risks and even more importantly to deal with them effectively when they occur. Transdisciplinary entities should be tasked with assessing systemic risks and proposing mechanisms to strengthen resilience according to national and local contexts.

 Fourth, the prominence of efficiency goals in many economic and social processes obfuscate what makes human societies thrive in normal times and even more in times of crisis. As we are living in a situation where prevailing systems are part of the problem, the challenge is to introduce transformative change in a way that is not excessively destructive. A complexity lens calls for developing policy goals and measurements that focus on buffer, redundancy, and spare capacity.

Overall, tackling complex global challenges such as the COVID-19 pandemic requires new forms of cross-disciplinary knowledge generation and integration.^22^ In addition to actions identified above, an urgent need is to better prepare the next generation in applying the complexity mindset. This requires the advancement of transdisciplinary global system science.

## Ethical issues

 Not applicable.

## Competing interests

 Authors declare that they have no competing interests.

## Authors’ contributions

 DW initiated and drafted the paper; FT, KB, KL, CM, DP, NL and OY provided content and edited the manuscript. DW produced the figure.

## Funding

 The project entitled “Governing systemic crises in the 21st century: Learning from early Covid-19 responses in Europe” is supported by the Swiss National Science Foundation (Grant 31CA30_196396).
